# Antimicrobial and Attractant Roles for Chemerin in the Oral Cavity during Inflammatory Gum Disease

**DOI:** 10.3389/fimmu.2017.00353

**Published:** 2017-03-29

**Authors:** Urszula Godlewska, Piotr Brzoza, Aneta Sroka, Pawel Majewski, Holger Jentsch, Martin Eckert, Sigrun Eick, Jan Potempa, Brian A. Zabel, Joanna Cichy

**Affiliations:** ^1^Faculty of Biochemistry, Biophysics and Biotechnology, Department of Immunology, Jagiellonian University, Kraków, Poland; ^2^Faculty of Biochemistry, Biophysics and Biotechnology, Department of Microbiology, Jagiellonian University, Kraków, Poland; ^3^Centre for Periodontology, Department of Cariology, Endodontology and Periodontology, University Hospital of Leipzig, Leipzig, Germany; ^4^Department of Periodontology, School of Dental Medicine, University of Bern, Bern, Switzerland; ^5^Department of Oral Immunology and Infectious Diseases, University of Louisville School of Dentistry, Louisville, KY, USA; ^6^Palo Alto Veterans Institute for Research, VA Palo Alto Health Care System, Palo Alto, CA, USA

**Keywords:** chemerin, antimicrobial peptides, chemoattractant, inflammation mediators, microbiota, oral cavity

## Abstract

Periodontal inflammation is one of the most common chronic inflammatory conditions in humans. Despite recent advances in identifying and characterizing oral microbiota dysbiosis in the pathogenesis of gum disease, just how host factors maintain a healthy homeostatic oral microbial community or prevent the development of a pathogenic oral microbiota remains poorly understood. An important determinant of microbiota fate is local antimicrobial proteins. Here, we report that chemoattractant protein chemerin, which we recently identified as a potent endogenous antimicrobial agent in body barriers such as the skin, is present in the oral cavity under homeostatic and inflammatory conditions. Chemerin and a chemerin-derived antimicrobial peptide are bactericidal against select bacteria strategically positioned in dental biofilm. Gingival crevicular samples from patients with gingivitis but not periodontitis contain abundant bioactive chemerin capable of inducing CMKLR1-dependent leukocyte migration. Gingipains secreted by the periodontopathogen *P. gingivalis* inactivate chemerin. Together, these data suggest that as an antimicrobial agent and leukocyte chemoattractant, chemerin likely contributes to antimicrobial immune defense in the oral cavity.

## Introduction

Chemerin is a chemoattractant protein implicated in recruitment of dendritic cells (DCs), macrophages, and NK cells to sites of inflammation (1). These immune cells express the G-protein-coupled signaling receptor CMKLR1, which mediates cell chemotactic responses to bioactive chemerin (2, 3). Chemerin circulates as an inactive isoform (chemS163) and needs to be proteolytically processed to display chemotactic potential. Bioactive chemerin lacking six amino acids in the C-terminus (chemS157) has been isolated from body fluids (1), and various serine and cysteine proteases of host and microbial origin can generate active chemerin by C-terminal processing *in vitro* (4–6). However, proteases can also inactivate or degrade the attractant and thus limit the extent of chemerin activity (1). Chemerin is broadly expressed in numerous anatomic sites, including liver and fat tissues as well as by epithelial cells in the skin epidermis (7–10), intestinal epithelium (8, 11), and pulmonary airways (8, 12). The strategic positioning of chemerin at the host-environment interface suggests a role in antimicrobial defense.

We recently demonstrated that human recombinant chemerin as well as endogenous chemerin secreted by primary human keratinocytes in organ cultures significantly inhibited growth of skin bacteria (6, 9). As is the case for chemoattractant activity, the inhibitory C-terminal peptide present in the chemerin holoprotein chem163S must be removed for full antibacterial effects. An internal 20-amino acid peptide V^66^–P^85^ embodies most of the antimicrobial activity of active chemerin, and is comparable in potency to other antimicrobial peptides (AMPs) (9). Chemerin was recently reported to be present in the oral cavity, and the levels of chemerin in saliva and gingival crevicular fluid were increased in patients suffering from periodontitis (13, 14). Since periodontitis is associated with an imbalance of oral microbiota (15, 16), these findings together suggest that chemerin might influence disease processes through controlling bacteria burden and/or composition in the oral cavity and by regulating immune cell infiltration.

The human oral cavity harbors diverse microbes that colonize gingiva and teeth (15, 17). The majority of bacteria in the oral cavity are organized in a biofilm structure. Primary (early) colonizers mostly belong to *Streptococcus* genera represented by *S. oralis, S. mitis*, and *S. sanguinis*, three of the most prevalent species of streptococci that are able to attach to enamel and epithelial surfaces (18). The streptococci co-aggregate with other bacteria species represented by many genera, including *Prevotella* spp. and *Actinomyces* spp. Since many primary colonizers lack specific adhesins required for coaggregation, assembly of a more diverse and larger community is promoted by specific bacterial species such as *Fusobacterium nucleatum*. Usually found in the middle layer of a biofilm, *F. nucleatum* coordinates the coaggregation of early and late colonizers (16). Notably, colocalization of *F. nucleatum* with so-called “red complex” pathogens, such as *Treponema denticola, Porphyromonas gingivalis*, and *Tannerella forsythia* (19), suggests that integration of these pro-inflammatory and tissue-destructive pathogens to dental plaque is *F. nucleatum* dependent (20–22).

The oral cavity, in common with other portals of microbe entry, contains a variety of AMPs that can restrict the growth of bacteria and prevent potential pathologic outcomes. Salivary glands, epithelial cells, and oral cavity-recruited neutrophils produce over 45 AMPs that are detected in saliva and gingival crevicular fluid. These include cathelicidins (LL37), α-defensins, β-defensins, histatins, and secretory leukocyte protease inhibitor (SLPI) (23–26). Because AMPs utilize different strategies to restrict microbial growth (24, 27), their diversity may be important for independently controlling the load, composition, and location of microbial communities in the oral cavity and for maintaining oral homeostasis.

Here, we report that chemerin directly acts on specific oral bacteria strains and exhibits chemotactic activity in gingivitis patient samples. Together, these findings suggest that antimicrobial and chemoattractant chemerin can shape the oral microbiome and coordinate oral immune defense mechanisms.

## Materials and Methods

### Materials

Chemerin peptide 4 (VRLEFKLQQTSCRKRDWKKP) (p4) and scramble peptide 4 (DPWLKVRKFQTLKQREKRCS) (scp4) were chemically synthesized by ChinaPeptide (Shanghai, China). Chemically synthesized LL37 was from Emory Microchemical Facility (Atlanta, GA, USA). Recombinant human full-length chemerin variant chem163S and chemerin variant chem157S, lacking 6 aa at C-terminus were produced in *Pichia pastoris*. DNA fragments corresponding to the desired chemerin proteins were amplified by PCR and cloned into the pPIC9K expression vector (Life Technologies, Carlsbad, CA, USA) using the overlap-extension PCR method (28). Both constructs lacked the 20 aa chemerin signal peptide but had the hexahistidine tag (His-tag) and the enterokinase cleavage site added to their N terminus. The identity of created constructs was verified by sequencing (Genomed, Poland). Recombinant chemerin isoforms were produced using *P. pastoris* strain GS115 (His^−^), transformed with the SalI-linearized pPIC9-chemerin construct. Recombinant proteins were purified from the supernatants of His + transformants using Ni-Sepharose 6 Fast Flow (GE Healthcare, Uppsala, Sweden). The recombinant chemerin variants were eluted with 500 mM imidazole and dialyzed against phosphate buffered saline (PBS). The molecular weight and purity of the obtained proteins was determined by SDS-PAGE and Coomassie Blue staining. Since the final recombinant chemerin variants contained a His-tag, we also evaluated in parallel a His-tag free, HPLC-purified chemerin variant lacking 16 aa at the C-terminus, chem147S (1, 5), with similar results (data not shown). Recombinant human SLPI was produced as previously described (29).

### Human Samples Collection

Unstimulated whole saliva samples were collected from healthy volunteers [*n* = 15, male:female 5:10; age 28.5 ± 4.8 (21–36)]. The participants rinsed their mouth with water before the collection. Saliva samples were collected 10 min later and centrifuged twice (10,000 × *g*, 5 min) to remove cellular debris and stored at −80°C until used. Six patients with gingivitis and 15 patients with periodontitis were recruited in a private dental practice. Demografic and clinical data of the patients are listed in Table 1. Subgingival plaque and tissue were sampled from the deepest site per quadrant (in case of the gingivitis patients from the mesio-buccal site of the first molar) by inserting each an endodontic paper-point (ISO 055) for 30 s into the pocket until resistance felt. Paper-points were pooled, transferred into a tube and RNAlater™ storage solution (Sigma-Aldrich) was added. Not earlier than 24 h later, gingival crevicular wash-out (GCF) was obtained as previously described (30). Immediately after sampling, paper-points were stored at −20°C and GCF samples at −80°C until assayed. The total protein concentration in saliva and GCF was determined by bicinchoninic acid assay (Sigma-Aldrich). The patients study protocol was approved by the Ethical Commission of the University of Leipzig. All subjects gave written informed consent in accordance with the Declaration of Helsinki.

**Table 1:** **Demographic and clinical data**.

	Gingivitis (*n* = 6)	Periodontitis (*n* = 15)
Age: mean (range)	46.3 (17–78)	63.9 (51–78)
Male/female (*n*)	1/5	5/10
Sites with PD ≥ 5 mm (*n*; mean ± SD)	0	10.7 ± 6.5
Sites with AL ≥ 5 mm (*n*; mean ± SD)	0.67 ± 1.03	23.1 ± 15.0
BOP (%; mean ± SD)	44 ± 15	39 ± 14
PI (%; mean ± SD)	62 ± 11	46 ± 20

*PD, probing depth; AL, attachment loss; BOP, bleeding on probing; PI, plaque index*.

### PCR

DNA and RNA was simultaneously extracted from the paper-points using innuPREP RNA/DNA mini kit (Analytic Jena AG) according to the manufacturer’s instruction. DNA was used for real-time PCR analysis of the major periodontopathogens as previously described (31). RNA was converted to cDNA using the GoScript™ Reverse Transcription System (Promega) according to the manufacturers’ instructions. qPCR was performed using GoTaq^®^ qPCR Master Mix (Promega) and primers specific for *P. gingivalis* proteases (32): *rgpA*, 5′-TATCCTTCGTGATGTGCGTG-3′, 5′-GCTGTAACGGGAGAAGCAAT-3′; *rgpB*, 5′-CATTCTCCTCTCTGTTGGGA-3′, 5′-CGTAGGGGATTTGATCAGGA-3′; *kgp*, 5′-TCAAGCAGT CGATGCAAGC-3′, 5′-ACTTGGGTCAGTTCTTGTCC-3′; and *sod*, 5′-AATTCCACCACGGTAAGCAC-3′, 5′-TTCTCGATGGACAGTTTGCC-3′; as well as human chemerin 5′-TGGAAGAAACCCGAGTGCAAA-3′, 5′-AGAACTTGGGTCTCTATGGGG-3′, and GAPDH, 5′-GACAGTCAGCCGCATCTTCT-3′, 5′-TTAAAAGCAGCCCTGGTGAC-3′. The relative gene expression normalized to sod (rgpA, rgpB, kgp) and GAPDH (chemerin) was calculated using the 2^−ΔΔCT^ method (10, 33, 34).

### ELISA

Chemerin levels in saliva and GCF samples were quantified by ELISA. Monoclonal mouse anti-human chemerin (R&D System) and biotin-labeled polyclonal goat anti human chemerin (R&D System) Abs were used to detect chemerin. HRP-labeled streptavidin (BD Pharmingen) was used to bind the biotinylated Abs. The plate was developed with 3,3′,5,5′-Tetramethylbenzidine substrate (TMB) (BD Biosciences), stopped using 0.18M sulfuric acid and measured at 450 nm. Alternatively, commercially available chemerin ELISA kit (R&D Systems) was used according to the manufacturer’s recommendations.

### Bacterial Strains and Culture Conditions

The bacterial strains used in this study were standard strains except Sov-A mutant with replacement of *sov* gene (PG0809/10) in *Porphyromonas gingivalis* W83 with a tetQ cassette (Tc 1 µg/ml, Sigma-Aldrich). *P. gingivalis* ATCC 33277 was cultured in Brain Heart Infusion broth (BHI) (Becton Dickinson) supplemented with yeast extract (5 g/l, Lab Empire), hemin (10 mg/l, Sigma-Aldrich), l-cysteine (0.25 g/l, Lab Empire), menadione (0.5 mg/l, Sigma-Aldrich), and defibrinated sheep blood (5% v/v). *P. gingivalis* W83 and *P. gingivalis* Sov-A were cultured in Scheadler broth (BTL) supplemented with menadione (0.5 mg/l), defibrinated sheep blood (5% v/v). *Prevotella intermedia* 17, *Fusobacterium nucleatum* ATCC 10953, *F. nucleatum* ATCC 25586 were cultured in Trypticase soy broth (TSB) (Sigma-Aldrich) supplemented with defibrinated sheep blood (5% v/v). *Tannerella forsythia* ATCC 43037 was cultured in TSB supplemented with hemin (5 mg/l), N-acetyl muramic acid (10 mg/l, Sigma-Aldrich) and defibrinated sheep blood (5% v/v). *Streptococcus salivarius* ATCC 7073, *S. sanguinis* ATCC 10556, *S. oralis* ATCC 35037, *S. gordonii* ATCC 10558 were cultured in BHI. *P. gingivalis, P. intermedia, F. nucleatum*, and *T. forsythia* were grown in an anaerobic atmosphere using a GasPak™ EZ Anaerobe Pouch System (BD). Streptococci were grown in a 5% CO_2_ atmosphere using a GasPak™ EZ CO_2_ Pouch System (BD).

### Microdilution Assay (MDA)

Overnight cultures of bacterial strains were sub-cultured to fresh media and grown to mid-logarithmic phase. Bacteria were harvested, washed three times with Dulbecco’s PBS and diluted to 4 × 10^5^ CFU/ml with PBS. Bacteria were then incubated for 3 h with either p4 at 100 µM or scp4 (negative control) at 100 µM. LL37 (50 µg/ml) was used as a positive control.

### Minimal Inhibitory Concentration (MIC) Determination

Minimal inhibitory concentration was determined as previously described (35) with some modifications. Briefly, bacteria were prepared as described above and diluted to 4 × 10^6^ CFU/ml with 0.2% bovine serum albumin in PBS. Series of twofold dilution of peptide p4 were prepared in PBS to provide final concentration range 400–0.4 µg/ml (154–0.15μM). Bacterial suspensions were mixed with p4 or PBS (control) and incubated at 37°C overnight. The MIC was defined as the lowest antimicrobial concentration which prevented visible growth of bacteria.

### Biofilm Formation

Overnight bacterial cultures were harvested, washed with PBS and adjusted to OD_600nm_ = 1. One hundred microliters of bacterial suspension previously diluted 1:100 in BTL supplemented with menadione (0.5 mg/l) were grown in flat-bottomed 96-well polystyrene microplates (Sarstedt) under anaerobic condition. After incubation for 48 h, supernatant containing planktonic cells and media was gently removed and replaced with p4 (100 µM) in PBS or PBS alone (control). The viability of the bacterial cells in biofilm after 5 h treatment with p4 was determined using The BacTiter-Glo™ Microbial Cell Viability Assay (Promega) based on ATP-based luminescence quantification. The number of viable bacteria was conducted by counting CFU/ml. Following the incubation with p4, biofilms were resuspended, serially diluted and plated on an agar medium.

### Protease Activity Assay

Overnight cultures of *P. gingivalis* W83 strain were adjusted to OD_600nm_ = 1 with BTL and centrifuged for 15 min at 6,000 × *g*. The conditioned media were harvested and stored at 4°C until used. Conditioned media and GCF samples were tested for protease activity using N_α_-Benzoyl-l-Ala 4-nitroanilide hydrochloride and N-(p-Tosyl)-Gly-Pro-Lys 4-nitroanilide acetate salt (both from Sigma) hydrolyzed by Rgp and Kgp gingipains, respectively. Ten microliters of bacterial conditioned media (corresponding to 10^7^ CFU) or 4 µl GCF samples (each containing 48 µg of total protein) were diluted with TNC buffer (200 mM Tris, 150 mM NaCl 5 mM CaCl_2_ pH = 7.6) with 10 mM l-cysteine to a final volume of 100 µl (for bacterial supernatants) or 50 µl (for GCF). To inhibit gingipains, 1 µM of either KYT–1 (RgpA and RgpB-specific inhibitor) or KYT–36 (Kgp-specific inhibitor) (36), were added. The mixtures were then preincubated for 20 min at 37°C. Equal volume of Rgp or Kgp substrate diluted in TNC buffer was then added to final substrate concentration of 1 mM and final volume of either 100 or 200 µl. The increase of absorbance at λ = 405 nm as a measure of hydrolyzed substrate was recorded for at least 90 min at 37°C, using a microplate reader (Tecan Infinite M200).

### Chemotaxis Assay

GCF samples from gingivitis and periodontitis patients were tested in an *in vitro* chemotaxis assay using 5 µm pore Transwell inserts (Costar) and murine pre-B lymphoma cell line L1.2 or L.1.2 cells stably transfected with human recombinant CMKLR1 (CMKLR1/L1.2). Chemotaxis assay was performed using chemotaxis media [RPMI 1640 w/l-glutamine supplemented with 25 mM HEPES (Biowest) and 10% FBS (Gibco)]. One hundred microliters of cells (2 × 10^5^ cells/well) were added to the top well and tested samples, each containing 1 nM of chemerin (as determined by ELISA) were added to the bottom well in a 600 µl volume. Migration was assayed for 2 h at 37°C. The inserts were then removed and cells that had migrated through the filter to the lower chamber were collected and counted by flow cytometry (LSRII, BD). The results are presented as% input migration.

### Chemerin Processing by Gingipains

Ten microliters of conditioned media derived from overnight cultures of *P. gingivalis* W83 (corresponding to 10^7^ CFU) were mixed with 5 µl of TNC buffer with 10 mM l-cysteine. For inhibition test, KYT-1 and KYT-36 were added to a final concentration of 1 µM. The samples were preincubated for 20 min at 37°C, followed by overnight incubation with 50 ng human recombinant chemS157. The samples were then analyzed in an *in vitro* chemotaxis assay.

## Results

To determine whether chemerin serves as AMP against oral bacteria, we first analyzed chemerin protein levels in saliva of healthy individuals with no signs of periodontal disease. In agreement with a recent report (13), chemerin was found in saliva from healthy donors, in the concentration of 1.3 ± 0.5 ng/mg total protein (mean ± SD, *n* = 15). Next, we determined chemerin RNA and protein levels in gingiva samples from patients suffering from periodontal inflammation. We analyzed samples from patients with either gingivitis (gum inflammation) or periodontitis, a more severe form of gum disease associated with progressive alveolar bone loss around the teeth. Donor demographics and clinical parameters are included in Table 1.

As anticipated, periodontitis patients presented with significantly elevated colony counts of the periodontopathogen *P. gingivalis* and its associated gingipain proteases (RgpA, RgpB, and Kgp) compared to patients with gingivitis (Table 2). Chemerin gene expression tended to be higher in periodontal region samples from the periodontitis group compared to gingivitis, although the difference was not statistically significant (Table 2). Likewise, GCF samples from gingivitis and periodontitis patients contained on average similar levels of chemerin (5.6 ± 1.3 ng/mg of total protein vs. 6.3 ± 1.1 ng/mg, mean ± SEM) and Table 2. While there were no significant correlations between chemerin protein levels and the examined clinical/microbiological variables (data not shown), there was a significant positive correlation between chemerin gene expression and clinical probing depth and attachment loss measurements as well as abundance of *P. gingivalis* and *T. forsythia* in the pocket samples (Table 3). Together these data suggest that chemerin may be involved in the regulation of microbiota in the oral cavity under both homeostatic and inflammatory conditions.

**Table 2:** **Microbiological and chemerin expression data in gingival samples from patients with peridontal disease**.

	Gingivitis *n* = 6		Periodontitis *n* = 15		
	Positive samples with bacteria loads ≥ 10^5^ (*n*/total)	Median [interquartile range] × 10^5^	Positive samples with bacteria loads ≥ 10^5^ (*n*/total)	Median [interquartile range] × 10^5^	*p* (Mann–Whitney)
*A. actinomycetemcomitans*	1/6	0.00 [0.00; 3.44]	7/15	0.00 [0.00; 2.97]	0.340
*P. gingivalis*	5/6	0.07 [0.01; 3.84]	13/15	1.03 [0.51; 2.10]	0.029*
*T. forsythia*	4/6	1.13 [0.00; 16.9]	15/15	1.66 [0.39; 33.3]	0.080
*T. denticola*	3/6	0.22 [0.00; 1.32]	6/15	0.00 [0.00; 0.11]	0.340

	**Positive samples (***n***/total)**	**Median [interquartile range] × 10^5^**	**Positive samples (***n***/total)**	**Median [interquartile range] × 10^5^**	

*rgpA* RNA relative to sod	1/6	0.00 [0.00; 0.45]	12/15	0.35 [0.02; 2.57]	0.023*
*rgpB* RNA relative to sod	1/6	0.00 [0.00; 3.16]	12/15	0.76 [0.22; 3.38]	0.045*
*kgp* RNA relative to sod	1/6	0.00 [0.00; 2.77]	12/15	0.52 [0.26; 158.3]	0.023*
Chemerin mRNA relative to GAPDH	5/6	0.33 [0.00; 1.63]	13/15	1.04 [0.31; 1.93]	0.205

	**Positive samples (***n***/total)**	**Median [interquartile range]**	**Positive samples (***n***/total)**	**Median [interquartile range]**	

Chemerin protein (ng/mg total protein)	6/6	4.37 [0.43; 17.84]	15/15	6 [2.81; 10.30]	0.340

*Statistically significant.

**Table 3:** **Correlation of chemerin gene expression and chemerin protein levels with clinical and microbiological variables (*P* and *R* are only given if *p* was statistically significant) Spearman Rho**.

	Chemerin expression	
	*R*	*P*
PD ≥ 5 mm (*n*)	0.472	0.031
AL ≥ 5 mm (*n*)	0.451	0.034
BOP (%)		n.s.
PI (%)		n.s.
*A. actinomycetemcomitans*		n.s.
*P. gingivalis*	0.462	0.035
*T. forsythia*	0.550	0.010
*T. denticola*		n.s.
Chemerin protein level		n.s.

*PD, probing depth; AL, attachment loss; BOP, bleeding on probing; PI, plaque index; n.s., not significant*.

**Figure 1:**
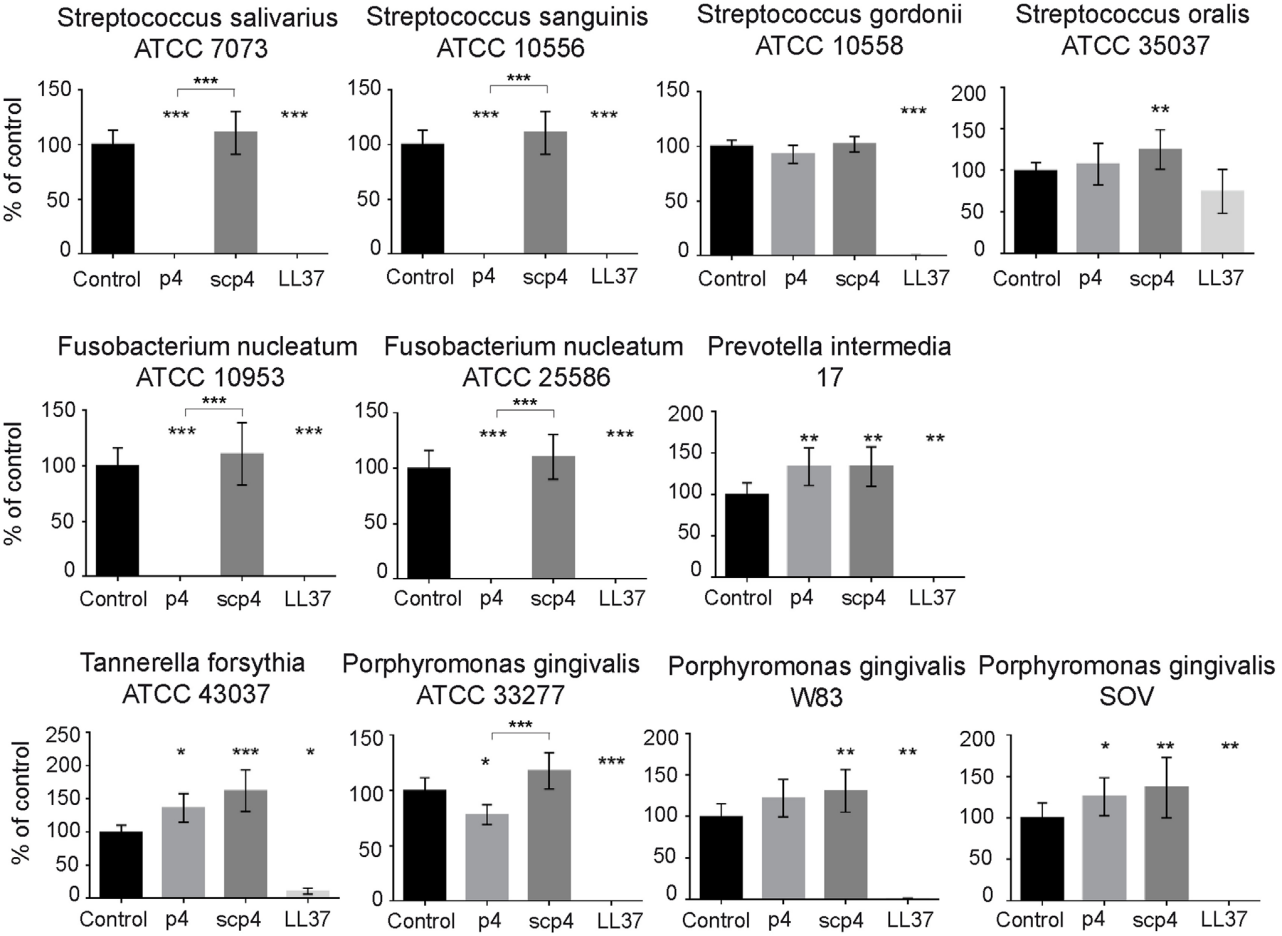
**Selective antimicrobial activity of chemerin peptide p4 against oral bacteria**. Chemically synthesized chemerin p4 and scramble p4 (scp4) peptides as well as LL37 were tested against the indicated oral cavity bacterial strains using MDA assay. Bacteria were incubated with p4 and scp4 at 100 µM, and LL37 at 11 µM for 3 h. The results are expressed as the mean ± SD of three independent experiments. ***p < 0.001, **p < 0.01, *p < 0.05 by Kruskal–Wallis one-way ANOVA with post Dunn’s test for multiple comparison, comparing vehicle treated bacteria (control) and the peptide-treated bacteria, or p4 vs. scp4.

**Figure 2:**
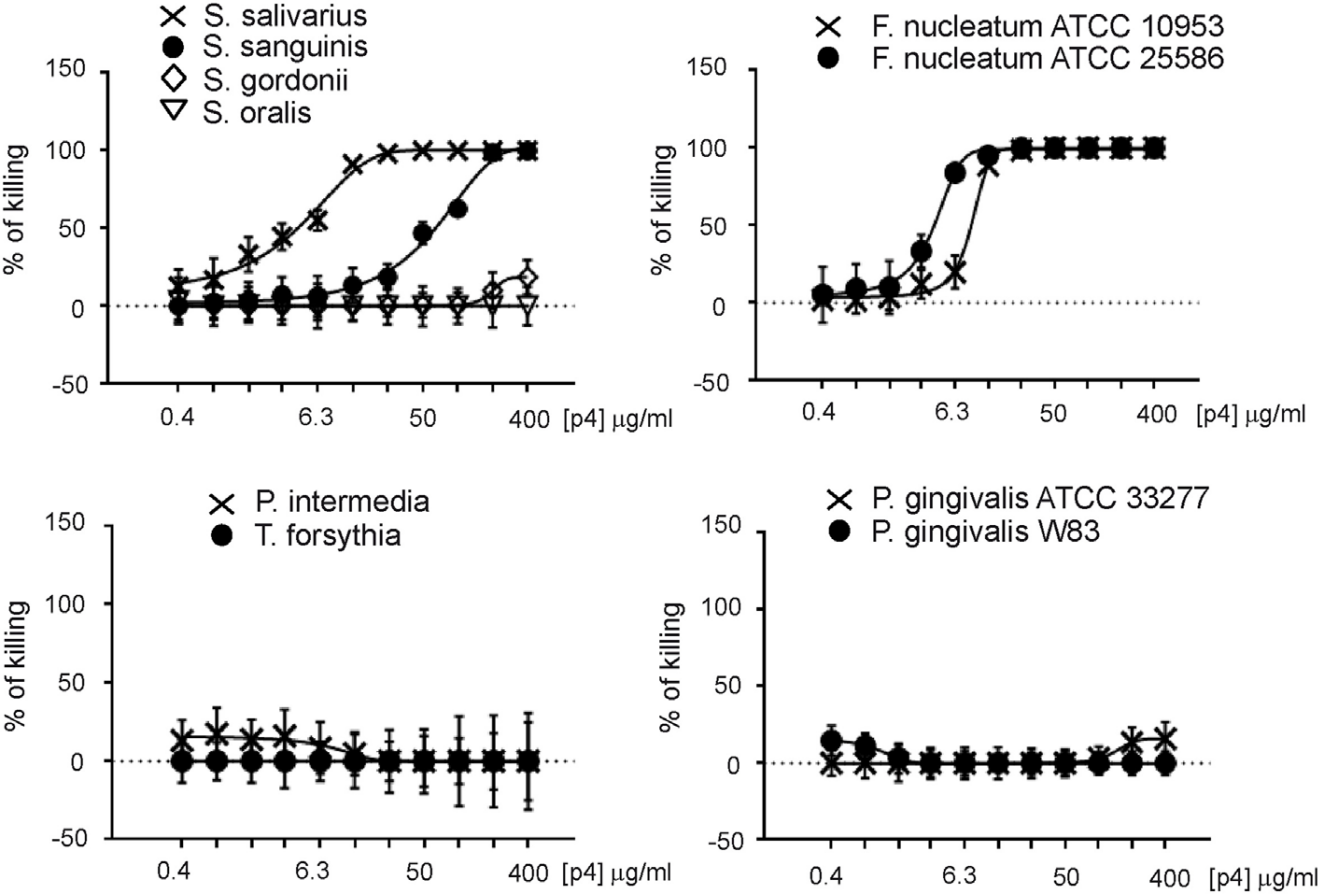
**Minimal inhibitory concentration (MIC) values for indicated oral cavity microorganisms**. Data indicate% of killing for the indicated strain. The MIC was defined as the lowest concentration of p4 showing no visible growth (100% of killing). Mean ± SD of three independent measurements is shown.

We next explored whether chemerin exhibits antimicrobial activity against selected oral bacterial species that are differently located in oral biofilm and differ in pathogenic potency. Since chemerin peptide 4 (p4), corresponding to the internal Val^66^–Pro^85^ region of human chemerin, is primarily responsible for chemerin antimicrobial effects (9), we tested selected components of the oral microbiota for sensitivity to p4 by MDA assay. This assay detects the planktonic (free floating) form of bacteria. Scramble peptide 4 (scp4, 100 µM) and oral antimicrobial agent LL37 (11 µM) (24), were used as a negative and positive controls, respectively. Chemerin peptide p4 (100 µM) completely inhibited the growth of two out of four tested *Streptococcus* species, *S. salivarius* ATCC 7073 and *S. sanguinis* ATCC 10556, but not *S. gordonii* ATCC 10558 or *S. oralis* ATCC 35037. In contrast, LL37 completely inhibited the growth of *S. gordonii* ATCC 10558 but not *S. oralis* ATCC 35037 (Figure 1). Peptide p4 also completely inhibited the growth of two different strains of *F. nucleatum*; *F. nucleatum* ATCC 10953 and ATCC 25586. However, the growth of *P. intermedia* 17 or the red complex constituent *T. forsythia (T. forsythia* ATCC 43037) was not inhibited by p4, although they were largely susceptible to LL37-dependent growth inhibition (Figure 1). Likewise, only slight but significant inhibition of another red complex component *P. gingivalis* was noted, leading to survival of 78 ± 9% of bacteria compared to the vehicle-treated *P. gingivalis* ATCC 33277 (set as 100%) or to the scramble p4 peptide control (118 ± 17%). The growth/viability of two other *P. gingivalis* strains (W83 and SOV) was not suppressed by p4, although LL37 significantly inhibited their growth (Figure 1). In contrast to p4, scp4 did not limit the growth of the tested strains, and in some cases even increased the number of viable counts of bacteria (Figure 1). The differential anti-microbial activity of p4 against oral bacteria was further demonstrated by MIC values, which were 50 µg/ml (19.2 µM) for the most sensitive *Streptococci* and *F. nucleatum* strains, and 400 µg/ml or more for less sensitive and resistant strains (Figure 2). We conclude that chemerin peptide 4 has the ability to limit the growth of specific oral cavity-resident bacteria, but exhibits variation in its ability to kill different species of oral microbes.

**Figure 3:**
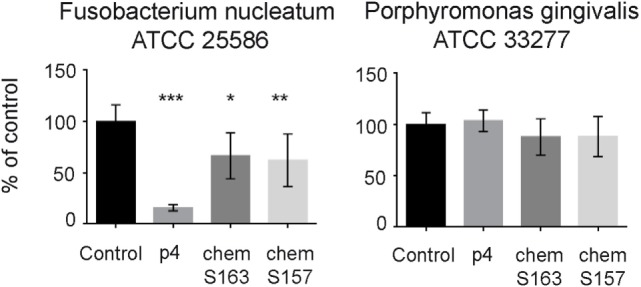
**Chemerin isoforms chemS163 and chemS157 exhibit antibacterial activity against *F. nucleatum* but not *P. gingivalis***. The antimicrobial activity of chemerin isoforms chemS163, chemS157 (both at 2µM), and p4 at 2µM were tested against *F. nucleatum* ATCC 25586 and *P. gingivalis* ATCC 33277 using MDA assay. The results are expressed as the mean ± SD of three independent experiments. ***p < 0.001, **p < 0.01, *p < 0.05 by Kruskal–Wallis one-way ANOVA with post Dunn’s test for multiple comparison, comparing vehicle treated bacteria (control), and the peptide-treated bacteria.

Next, we tested whether chemerin isoforms, either full-length, chemotactively inert chemS163, or the truncated, chemotactively active chemS157 (both containing the internal p4 sequence) affect the growth of p4-sensitive *F. nucleatum* and largely resistant *P. gingivalis*. Both chemS163 and chemS157 significantly suppressed the growth of *F. nucleatum* ATCC 25586 at 2µM (67 ± 23 and 62 ± 26% viability, mean ± SD, compared with control); however, their inhibitory effects were less robust than 2µM p4 (16 ± 3%) (Figure 3). On the other hand, neither chemerin isoforms showed significant antimicrobial activity against *P. gingivalis* ATCC 33277 (Figure 3). These data suggest that not only chemerin-derived peptide 4 but also full-length chemerin and its endogenous bioactive isoform inhibit the growth of selected oral bacteria.

**Figure 4:**
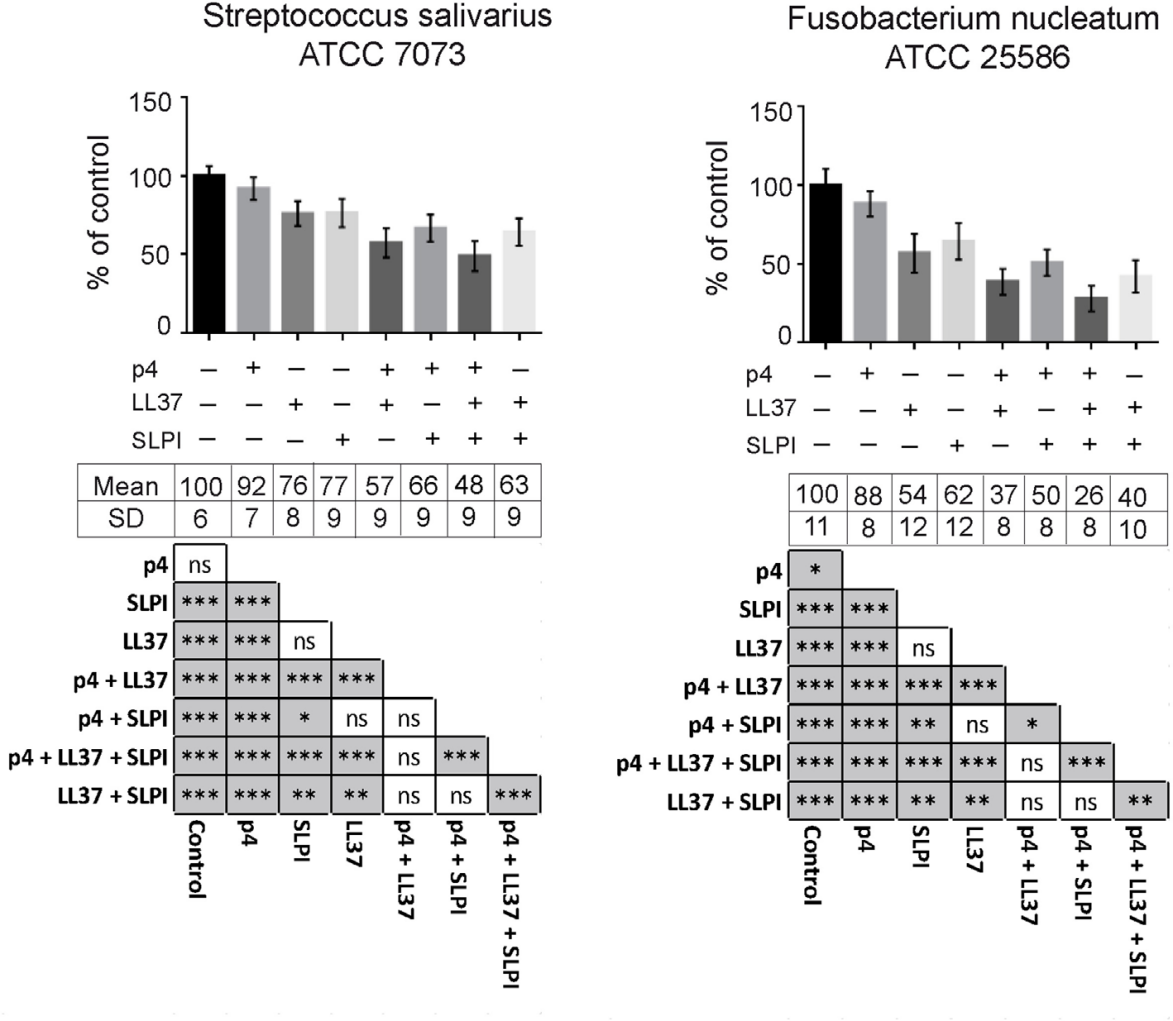
**Peptide p4 cooperates with LL37 and secretory leukocyte protease inhibitor (SLPI) in limiting growth of specific oral bacteria**. Chemically synthesized peptides, p4 and LL37 as well as recombinant human SLPI were tested against indicated oral cavity bacterial strains using MDA assay. Bacteria were incubated with 2.5 µg/ml p4 and LL37, and 10 µg/ml SLPI for 3 h. The results are expressed as the mean ± SD of three independent experiments. *p < 0.05, **p < 0.01, ***p < 0.001, ns, not significant by Kruskal–Wallis one-way ANOVA with post Dunn’s test for multiple comparison.

To evaluate potential additive or synergistic effects between chemerin and other oral AMPs, we next tested the antimicrobial activity of combinations of p4 with LL37 and SLPI. AMPs were tested at suboptimal concentrations that resulted in >30 and <90% growth inhibition when applied alone, based on previously determined MIC values for each AMP (Figure 2 and data not shown). Chemerin peptide p4 (2.5 µg/ml) given in combination with LL37 (2.5 µg/ml) and/or SLPI (10 µg/ml), in each case was significantly more bactericidal against *S. salivarius* and *F. nucleatum* than each agent given alone (Figure 4). These data suggest that all three AMPs cooperate for optimal restriction of bacterial growth in oral cavity.

**Figure 5:**
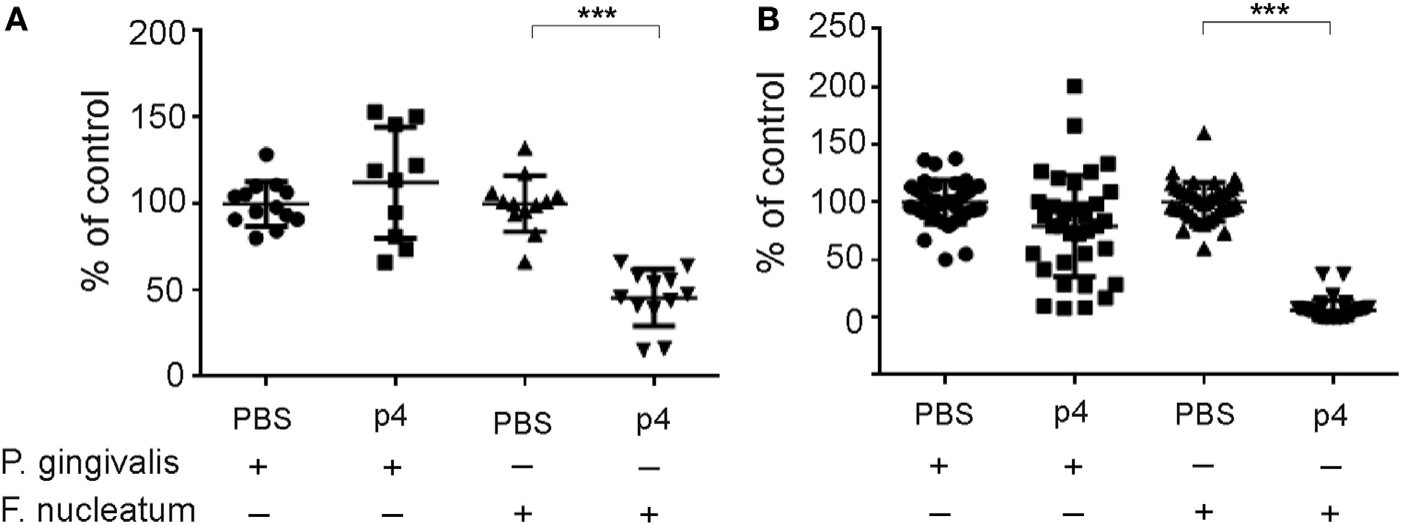
**Peptide p4 suppresses growth of *F. nucleatum* in biofilm *in vitro***. *F. nucleatum* ATCC 25586 and *P. gingivalis* W83 were grown attached in polystyrene microplates for 48 h followed by the incubation with 100 µM p4 or vehicle (PBS) for 5 h. The viability of bacteria was then determined by ATP-based luminescence quantification **(A)** or CFU counting **(B)**.The results are shown as % of control (vehicle treated bacteria) and are expressed as the mean ± SD of five independent experiments. ***p < 0.001, by *t* test, comparing vehicle vs. p4 for each bacteria.

The majority of bacteria in the oral cavity grow attached to the teeth and epithelial surfaces as biofilm components. Therefore, we next determined whether p4 inhibits the attached forms of *F. nucleatum* and *P. gingivalis*, in addition to suppressing the planktonic form of the bacteria (Figure 1). *F. nucleatum* and *P. gingivalis* were cultured for 48 h under biofilm-like conditions and then treated with p4 or vehicle PBS for 5 h. The growth of the attached form of *F. nucleatum* was markedly suppressed by p4 (Figure 5). In contrast, *P. gingivalis* was resistant to p4 treatment (Figure 5). These data suggest that chemerin exerts antimicrobial activity during either biofilm or planktonic life cycles of *F. nucleatum* but is minimally or not effective against planktonic and biofilm form, respectively, of *P. gingivalis*.

**Figure 6:**
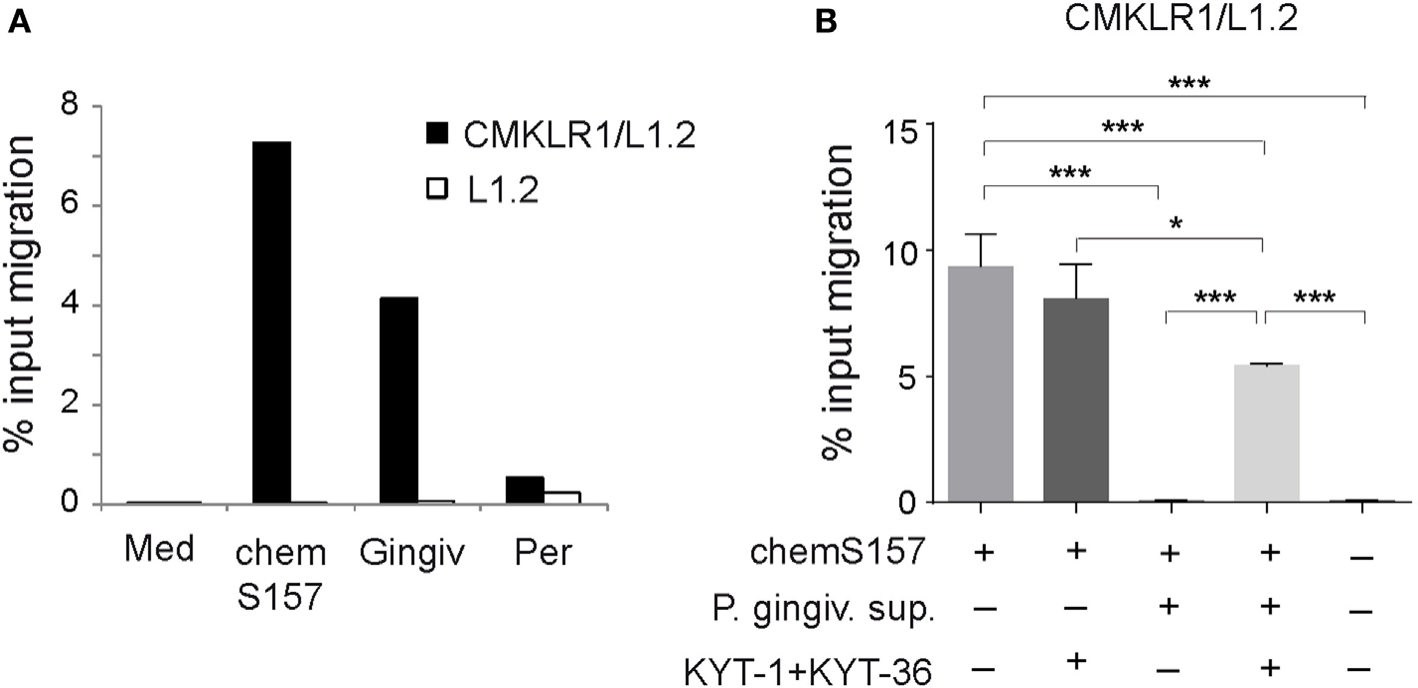
**GCF samples from gingivitis and periodontitis patients differ in chemerin-mediated chemotactic activity, and bioactive chemS157 is inactivated in gingipain-specific manner**. **(A)** Chemotactic bioactivity of endogenous chemerin from gingivitis (Gingiv) and periodontitis (Per) samples, each containing 1nM endogenous chemerin as determined by ELISA was evaluated by *in vitro* CMKLR1/L1.2 cell or L1.2 parental cell migration. Data are from one experiment, showing two combined samples in each patient group, and are representative of two experiments and five patients in each group. Migration to bioactive recombinant chemS157 at 1nM, and chemotaxis medium (Med) is shown as a positive and negative control, respectively. **(B)** 1nM recombinant chemS157 was incubated with the conditioned media from *P. gingivalis* W83 (*P. gingiv*. sup). Where indicated the media were first treated with gingipain-specific inhibitors (KYT-1 + KYT-36), followed by incubation with chemS157. The samples were tested in chemotaxis using CMKLR1/L1.2 transfectants. The mean ± SD from three experiments is shown. ***p < 0.001, *p < 0.05 by one-way ANOVA with *post hoc* Tukey Unequal N HSD test.

Since chemerin is chemoattractant for immune cells that are implicated in the pathogenesis of periodontal disease, including DCs, macrophages, and NK cells (37–41), we next tested whether chemerin in GCF samples from individuals with gingivitis and periodontitis is able to support chemotaxis of chemerin responsive CMKLR1^+^ cells. We performed *in vitro* transwell chemotaxis assays using the GCF samples each containing 1 nM endogenous chemerin (as determined by ELISA) and 1 nM bioactive chemS157 as a positive control. CMKLR1-transfected L1.2 cells migrated to GCF from gingivitis patients (4.2%) but showed little migration to GCF from periodontitis individuals (0.5%) (Figure 6A). When CMKLR1-negative parental L1.2 cell were used instead of CMKLR1/L1.2 transfectants, very little chemotaxis was detected (Figure 6A). These data indicate that the observed response was chemerin-dependent.

Since GCF samples from the gingivitis and periodontitis groups contained similar levels of chemerin (Table 2) but markedly differed in the attractant activity (Figure 6A), these data suggest that chemerin is inactivated in periodontitis patients, possibly by factors secreted by periodontal pathogens. The possible candidates include *P. gingivalis*-produced gingipains (RgpA, RgpB, and Kgp) (Table 2). To determine whether these proteases inactivate chemerin, *P. gingivalis* conditioned media were incubated with recombinant chemS157. The involvement of gingipains in chemerin inactivation was examined using conditioned media in which these enzymes were blocked by gingipain-specific inhibitors prior to incubation with chemS157. As shown in Figure 6B, CMKLR1-transfected L1.2 cells migrated robustly in response to chemS157, but almost no migration was noted when chemS157 was treated with *P. gingivalis* conditioned media. The effect of the conditioned media was largely reversed by gingipain-specific inhibitors, indicating that gingipains inactivate the chemoattactant activity of chemerin.

## Discussion

Chemerin is expressed by many different epithelial tissues and plays a role in skin immune defense (1, 9, 10). Here, we demonstrate that chemerin is also expressed in the oral cavity, where it likely contributes to antibacterial defense as an antibacterial agent and as a chemoattractant for immune cells. Using human samples, we showed that chemerin mRNA and protein is present in tissue cavity samples of individuals with inflammatory gum disease, and that chemerin protein is present in the saliva of healthy donors. Both planktonic and attached cultures of bacteria that specifically inhabit the oral cavity were sensitive to chemerin-derived peptide-p4 and chemerin protein isoforms. Thus, the physiological role of chemerin in the oral cavity might be to directly mold the microbiome. Furthermore, chemerin present in GCF from gingivitis patients retained leukocyte chemoattractant activity rendering it uniquely suited to position DCs, macrophages, and/or NK cells to sites of gum inflammation, where they may provide immune protection.

Our findings add chemerin to the list of potential endogenous oral AMPs. However, in contrast to other antimicrobial agents, such as LL37, chemerin exhibited a rather narrow spectrum of activity against oral microbiota (Figures 1 and 2). Among inhabitants of the periodontal biofilm, only *Streptococcus* species and most notably *F. nucleatum* strains were identified as p4 targets. Several *P. gingivalis* strains, including SOV were not inhibited by p4. Given that the *P. gingivalis* SOV strain is defective in secretion of gingipains (42), these data suggest that that resistance of *P. gingivalis* to p4 is not due to degradation of p4 by these enzymes. This differential ability of either p4 or chemerin to inhibit the growth of oral bacterial strains suggests that chemerin is not a direct chemical shield against pathogens but rather serves to shape the oral microbial ecology. By acting on a defined spectrum of microbes, chemerin might limit an assembly of a disease-provoking microbiota. For example, the presence of chemerin-sensitive *F. nucleatum* benefits the entire community of bacterial inhabitants in subgingival plaque due to its unique ability to cooperate with other bacterial species in biofilm formation (16). Recent reports associate periodontal pathogenicity with an imbalance in microbiota, known as dysbiosis (15, 43, 44). Therefore, through curtailing expansion of *F. nucleatum*, chemerin may help to maintain balance in the resident microbial community.

In common with other AMPs, chemerin in saliva or the GCF samples was found in less-than-effective concentrations to exert bactericidal effect on its own [Table 2; Figure 2; (13, 14, 24)]. Since chemerin levels at oral infection sites are likely to be higher than in CGF wash outs, local chemerin may be present in sufficient quantities to control oral microbiota by itself. However, chemerin may be more effective as an oral AMP by acting in concert with other antimicrobial agents. This is in-line with our findings showing additive inhibitory effects of p4, LL37, and SLPI on the growth of oral bacteria.

Certain AMPs serve a second role in host defense as leukocyte attractants, and chemerin has the capacity to play a similar dual role in the oral cavity. Chemerin-mediated chemotaxis, exhibited by the gingivitis samples (Figure 6A), may serve to enhance the recruitment of CMKLR1^+^ cells to inflamed gums. CMKLR1+ DCs, macrophages, and NK cells have been associated with inflammatory gum disease and reported to infiltrate the inflamed gingiva (37–41). Although their role in gingivitis or periodontitis is not well defined, they are considered to be a part of the defense mechanism against microbial challenge in dental biofilm (37–41). However, since the innate immune response to microbes can lead to excessive inflammation and its associated damaging effects on healthy host tissue, these cells may also contribute to the pathogenesis of periodontal disease (37–41). In contrast to gingivitis, periodontitis is associated with a marked reduction in chemerin bioactivity (Figure 6A). These data suggest that the chemoattractant is locally functionally compromised in patients with periodontitis. Several lines of evidence support the role for gingipains in chemerin inactivation: (1) mRNA encoding all three gingipains (RgpA, RgpB, and Kgp) was present in higher levels and in a larger number of patients with periodontitis compared with individuals with gingivitis (Table 2); and (2) *P. gingivalis* supernatants inactivated chemS157 in the gingipain-dependent manner (Figure 6B). However, in contrast to *P. gingivalis* conditioned media, we were not able to detect gingipain-specific enzymatic activity in GCF samples from either gingivitis or periodontitis individuals, which may be related to the substantial dilution (100–200×) of the wash samples (data not shown). Nevertheless, these data suggest that chemS157 is a novel gingipain substrate and that periodontal pathogens may utilize gingipains to subvert chemerin-dependent antimicrobial defense mechanisms.

Previous studies reported significantly higher levels of chemerin in saliva from periodontitis patients compared with gingivitis patients and healthy controls (13), and in GCF samples from periodontitis patients compared with healthy controls (14). Although in our studies we did not directly compare chemerin levels in saliva or GCF samples within the same groups of patients, chemerin levels were similar in GCF samples from periodontitis and gingivitis patients, with a trend for highler levels in the periodontitis group (Table 2). Chemerin is subject to posttranslational regulation by proteolytic processing, and chemerin protein levels might be altered by degrading proteolytic enzymes present in inflamed tissues. Likewise, local degradation of chemerin by proteases such as gingipains in samples from periodontitis patients might also explain why, despite a positive correlation between chemerin gene expression in the pocket samples and the clinical and microbial variables, chemerin protein levels were not similarly correlated (Table 3).

Increased levels of bioactive chemerin are present in gingivitis patient samples as opposed to periodontitis and thus are associated with less-severe gum disease. This broadly supports a protective role for chemerin signaling in the oral cavity. One of the possible mechanisms underlying bioactive chemerin-mediated protection might involve production of antimicrobial agents by the infiltrating cells. Macrophages as well as NK cells are well-known to produce a variety of bactericidal compounds, including AMPs (45), and these agents might play an important role in containing infection. On the other hand, chemerin inactivation might help to limit excessive inflammation and tissue destruction, including bone loss observed during periodontitis. This is supported by findings that chemerin is a negative regulator of bone formation. Chemerin or CMKLR1 knock down in bone marrow-derived osteoblast precursor cells is associated with osteoblastogenesis (46). Since an imbalance between bone-forming osteoblast and bone-resorbing osteoclast is the underlying cause of bone loss in periodontitis (47), disruption of chemerin-mediated signaling may promote osteoblast development.

In summary, chemerin has the ability to directly and selectively destroy oral microbes and may therefore influence the incidence or progression of gum disease *via* altering the composition of the oral microbiome. In addition, through guiding immune cells to infection sites, chemerin may help to translate the signals of microbial insult to a host physiological response. Inactivation of chemerin in periodontal lesions might lead to the immune dysregulation. Alternatively, chemerin inactivation might represent a mechanism to suppress deleterious inflammation and bone loss that characterizes periodontitis.

## Author Contributions

Conceived and designed experiments: UG, PB, AS, SE, JP, and JC; performed the experiments: UG, PB, AS, and ME; contributed reagents/materials: PM, BZ, HJ, and ME; wrote the paper: JC and BZ.

## Conflict of Interest Statement

The authors declare that the research was conducted in the absence of any commercial or financial relationships that could be construed as a potential conflict of interest.
